# Asthma and COVID-19 Associations: Focus on IgE-Related Immune Pathology

**DOI:** 10.3390/life12020153

**Published:** 2022-01-20

**Authors:** Chung-Jen Wang, Shih-Lung Cheng, Sow-Hsong Kuo

**Affiliations:** 1 Department of Internal Medicine, Far Eastern Memorial Hospital, New Taipei City 22056, Taiwan; dr.chung.jen.wang@gmail.com (C.-J.W.); shihlungcheng@gmail.com (S.-L.C.); 2 Department of Chemical Engineering and Materials Science, Yuab Ze University, Taoyuan City 32003, Taiwan

**Keywords:** asthma, COVID-19, biologics, IgE, omalizumab

## Abstract

Management of patients with asthma during the coronavirus disease 2019 (COVID-19) pandemic is a concern, especially since asthma predisposes patients to respiratory problems. Interestingly, asthma characterized by type 2 inflammation, also known as T-helper type 2-high endotype, displays a cellular and molecular profile that may confer protective effects against COVID-19. The results of experimental and clinical studies have established the actions of immunoglobulin E (IgE) in inducing airway hyperreactivity and weakening an interferon-mediated antiviral response following respiratory viral infection. Robust evidence supports the beneficial effect of the anti-IgE biologic treatment omalizumab on reducing respiratory virus-induced asthma exacerbations and reducing the frequency, duration, and severity of respiratory viral illness in patients with asthma. Indeed, accumulating reports of patients with severe asthma treated with omalizumab during the pandemic have reassuringly shown that continuing omalizumab treatment during COVID-19 is safe, and in fact may help prevent the severe course of COVID-19. Accordingly, guidance issued by the Global Initiative for Asthma recommends that all patients with asthma continue taking their prescribed asthma medications, including biologic therapy, during the COVID-19 pandemic. The impact of biologic treatments on patients with asthma and COVID-19 will be better understood as more evidence emerges.

## 1. Introduction

Globally, the coronavirus disease 2019 (COVID-19) pandemic has inflicted enormous health and societal impact, and will likely continue to do so into the foreseeable future. Not only has COVID-19 directly caused morbidity and mortality at historic levels, it has also been attributed to significant consequences in the management of patients with chronic diseases. COVID-19 is caused by the novel severe acute respiratory syndrome coronavirus 2 (SARS-CoV-2), and can lead to respiratory failure and death, similar to novel coronavirus diseases that have occurred in the past, such as the severe acute respiratory syndrome (SARS) and the Middle East respiratory syndrome (MERS) [1]. Understandably, there has been concern that patients with chronic respiratory diseases, such as asthma, may be at an increased risk of poorer outcomes if infected with SARS-CoV-2, which has led to considerations of adjustments to the standard management of these patients during the pandemic. Fortunately, clinical data accumulated thus far have revealed that people with asthma do not seem to suffer a markedly increased risk of SARS-CoV-2 infection or burden from COVID-19 compared to people without asthma [2,3,4,5]. However, as the manifestation of COVID-19 clearly shows a high degree of variation among those affected, it can still be reasonably expected that COVID-19 may have a variable impact across patients with different asthma types. Specifically, it has been hypothesized that the cellular and molecular profile of type 2 inflammation confers to a reduced susceptibility to COVID-19 in patients exhibiting this asthma endotype [6,7]. The immunoglobulin E (IgE) blocking agent omalizumab is a biologic therapeutic agent widely used as an adjunctive treatment in patients with persistent moderate-to-severe asthma, and markers of type 2 inflammation are used as predictors of anti-IgE treatment response [8]. However, the effects of immunomodulation with anti-IgE biologic treatment on COVID-19 have not been fully elucidated. With the aim of improving the understanding of the relationship between asthma, COVID-19, and asthma treatments including anti-IgE biologic therapy, current evidence regarding plausibly linked underlying mechanisms and potential implications in the management of patients with asthma during the COVID-19 pandemic are explored in this review.

## 2. Asthma Classification into Phenotypes and Endotypes

In all asthma patients, the disease manifests as an obstruction of airflow due to airway hyperresponsiveness, leading to symptoms of wheezing, shortness of breath, cough, and chest tightness. However, recent revelations in pathophysiology of asthma have uncovered underlying heterogeneity, and “asthma” is no longer considered a single disease entity but rather an umbrella term used to describe a collection of various phenotypes and endotypes [9]. While phenotyping categorizes asthma patients according to observable clinical characteristics, endotyping differentiates patients according to disease mechanisms at a molecular level, and thereby serves as an essential basis for an individualized treatment in precision medicine [10]. Traditionally, phenotyping of asthma has grouped patients into the broad categories of atopic/extrinsic asthma and non-atopic/intrinsic asthma based on clinically observable variables such as age at onset, exacerbating factors, concomitant comorbidities, and response to treatment [11,12,13]. However, experience has revealed that traditional phenotyping does not adequately reflect the diversity of the disease nor account for the varying responses to therapies [9,13]. Urged by the advent of a multitude biologic therapies that target specific inflammatory mediators, a shift toward an endotype-driven asthma treatment paradigm has occurred in recent years.

Based on the landmark study conducted by Wenzel et al. over 20 years ago, the most widely recognized inflammatory endotypes of severe asthma are currently the T-helper type 2 (Th2)-high endotype and Th2-low endotype [12]. The Th2-low endotype is characterized by neutrophilic or paucigranulocytic non-allergic airway inflammation associated with the elevation of the inflammatory mediators interleukin (IL)-1β, IL-6, IL-17, interferon (IFN)-ɣ, and tumor necrosis factor (TNF)-α [14]. In the Th2-high endotype, Th2 cells generate high levels of IL-4, IL-5, and IL-13, which drive IgE production and the recruitment of eosinophils. More recently, evidence has shown that group 2 innate lymphoid cells (ILC2s) are also producers of Th2-related cytokines in airway tissues and contribute to the initiation and propagation of airway inflammation [15,16]. Concordantly, Th2-high inflammation is also termed type 2 (or T2) inflammation to reflect that both adaptive immunity and innate immunity play important roles in asthma pathophysiology [9]. Although the presence of type 2 inflammation is more accurately determined by an assessment of key cellular effectors via tissue biopsy and induced sputum analysis, these invasive procedures are infeasible in routine clinical practice and are generally reserved for research [10]. Alternatively, measurement of fractional exhaled nitric oxide (FeNO), free and total IgE concentrations in serum, and eosinophil count in blood and sputum have been linked to type 2 cytokine involvement, and these biomarkers are currently utilized to approximately predict responsiveness to type 2 inflammatory pathway-targeted biologic therapies, including omalizumab (anti-IgE agent); mepolizumab, reslizumab, and benralizumab (anti-IL-5/IL-5 receptor agents); and dupilumab (anti-IL-4/IL-13 agent) [8].

## 3. Asthma, COVID-19, and ACE2 Interrelationship

Compared to individuals without asthma, patients with asthma are known to be at an increased risk for common viral respiratory infections as well as at an increased risk for infection-related complications such as virus-induced asthma exacerbations and viral respiratory infection requiring intensive care [17,18,19]. The United States Centers for Disease Control and Prevention has cautioned that people with moderate-to-severe asthma may be at an increased risk for SARS-CoV-2 infection and more severe COVID-19 [20]. Interestingly, however, asthma has not been consistently shown to be a clear risk factor for SARS-CoV-2 infection, and in fact, most studies have reported similar if not lower rates of asthma among patients with COVID-19 compared to the general population [21,22,23,24]. Asthma has also not been consistently shown to be a risk factor for severe clinical outcomes of COVID-19 [23,24,25,26]. A review of 150 studies conducted worldwide found comparable prevalence rates of asthma between patients with COVID-19 who were hospitalized vs. not hospitalized between patients with severe COVID-19 vs. not severe COVID-19, and between patients who died of COVID-19 vs. those who survived [23]. Furthermore, reported rates of hospitalization for COVID-19-related asthma exacerbations and asthma exacerbation during hospitalization for COVID-19 have been low [4,27].

**Figure 1:**
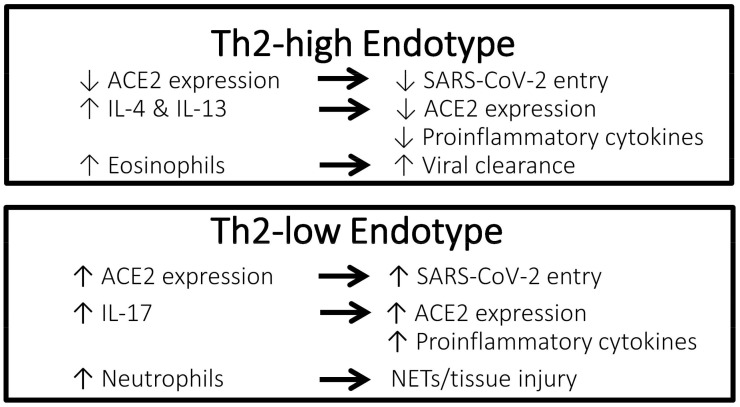
Characteristics of the Th2-high endotype asthma vs. Th2-low endotype asthma that may confer different effects against COVID-19. ACE2, angiotensin-converting enzyme 2 receptor; COVID-19, coronavirus disease 2019; IL, interleukin; NETs, neutrophil extracellular traps; SARS-CoV-2, severe acute respiratory syndrome coronavirus 2; Th2, T-helper type 2.

A possible protective effect of a Th2-high asthma endotype against poor clinical outcomes of COVID-19 has been suggested (Figure 1). A nationwide study in Korea showed that among patients with asthma, those with allergic asthma were at a lower risk of COVID-19 morbidity and mortality than patients with non-allergic asthma [24]. According to experimental studies, eosinophils may have a role in promoting respiratory virus clearance and antiviral host defense, which leads to the postulation that asthma patients with type 2 inflammation characterized by an increased number of eosinophils in the airway might be protected against severe COVID-19 outcomes [28,29]. In a retrospective cohort study conducted in Wuhan, China including 59 patients with confirmed COVID-19 and underlying chronic respiratory disease (chronic bronchitis, chronic obstructive pulmonary disease, or asthma), 73% of patients suffering severe COVID-19 had a low blood eosinophil count of less than 0.2 × 10^9^ cells/L compared to 24% of patients who had non-severe COVID-19 (*p* < 0.001) [30]. In a retrospective study including 951 patients with asthma and COVID-19 conducted in the United States, pre-existing eosinophilia defined as a blood eosinophil count of ≥150 cells/µL was associated with reduced COVID-19-related hospitalization and mortality [6]. In the same study, among patients who had eosinopenia (absolute eosinophil count of 0 cells/µL) at the time of hospitalization, those in whom the eosinophil count increased to above ≥150 cells/µL during admission were significantly less likely to die (mortality rate 9.6%) compared to patients whose eosinophil count remained <150 cells/µL throughout hospitalization (mortality rate 25.8%) (odds ratio [OR], 0.006; 95% confidence interval [CI], 0.0001–0.64; *p* = 0.03). In contrast, Th2-low endotype of asthma is characterized by neutrophilia, which is associated with a neutrophil extracellular trap formation and promotion of tissue injury, as well as an increased level of IL-17, which results in the propagation of proinflammatory cytokines [31].

Rhinoviruses are picoronaviruses that gain entrance into host airway epithelial cells via intracellular adhesion molecule 1 (ICAM-1), a low-density lipoprotein receptor (LDLR), and cadherin-related family member 3 (CDHR3) [32,33]. In patients with chronic allergic asthma, epithelial barrier disruption may lead to increased accessibility of rhinoviruses to CDHR3, which is mainly localized on cell surfaces along intercellular junctions [33]. In contrast, angiotensin-converting enzyme-2 (ACE2) has been identified as the receptor for the SARS-CoV-2 spike protein that provides the entryway for the virus into host cells [34]. Studies have reported a correlation between increased ACE2 expression and increased infectivity of SARS-CoV-2 [35,36]. Compared to airway cells of patients with non-allergic asthma, cells of patients with allergic asthma show lower ACE2 expression [7]. Exposure to the type 2 cytokines IL-4 and IL-13 was shown to reduce ACE2 expression in airway epithelial cells [37]. Furthermore, using a blood eosinophil count as a biomarker for type 2 inflammation, cut-off values of 150 and 300 cells/µL effectively identified patients with differential expression levels of ACE2 [38]. Therefore, evidence suggests that type 2 inflammation characteristic of the Th2-high asthma endotype is associated with lower ACE2 expression in the airway, thus potentially conferring a protective effect against COVID-19. Conversely, ACE2 expression has been shown to be upregulated by IL-17, which is elevated in the Th2-low asthma endotype [39].

## 4. Role of Airway Epithelium in COVID-19 and Asthma

Previously, airway epithelium was considered to simply serve as a mechanical barrier enabling gas exchange. It is now understood that airway epithelium is a complex tissue that performs a multitude of crucial functions, including the mediation of immune mechanisms [40]. The sinonasal airway epithelium is the initial site of SARS-CoV-2 infection and viral replication, and at this stage of infection, ciliated and mucus-secreting goblet cells that express ACE2 are the primary targets [41]. Early stages of COVID-19 are typically associated with relatively mild symptoms, likely related to the dampening of interferon-driven innate immune response to SARS-CoV-2 in nasal and bronchial epithelium [42,43]. Following the initial stage, the disease extends down the respiratory tract to the gas exchange portion of the lung where ACE2-expressing alveolar type II cells become the primary target of viral entry and replication [41]. Whereas in the nasal epithelium, damaged ciliated and secretory cells are replaced by progenitor basal cells that are spared from viral destruction [44], damage to alveolar type II cells results in much more dire consequences. Not only do alveolar type II cells secrete functional surfactant, they are also the progenitor cells for epithelial cells, and their destruction leads to gas exchange dysfunction, alveolar flooding from disrupted epithelium, and initiation of an innate immune response which further propagates alveolar damage due to inflammation [41].

In the context of asthma, the coordinated protective mechanisms of airway epithelium are disturbed differently in Th2-high (type 2 inflammation) and Th2-low (non-type 2 inflammation) asthma endotypes/phenotypes. The dysfunction of ciliated and secretory cells is observed in both asthma types; however, in Th2-high asthma the key early activators released by epithelial cells in response to allergens are IL-25, IL-33, and thymic stromal lymphopoietin (TSLP), whereas in Th2-low asthma, TNF-α, IL-6, IL-8, and IL-1β are the main inflammatory mediators released in response to environmental factors [45]. Importantly, in both asthma types, viruses are among the external factors that can instigate asthma pathogenesis at the epithelium, likely in part due to epigenetic mechanisms by which gene expression is modulated by DNA methylation, histone modulation, or translation modification by microRNAs in response to external stimuli [40,46]. How all of these factors are influenced by COVID-19 requires further study.

## 5. Therapeutic Management of Asthma Patients during the COVID-19 Pandemic

As asthma has not been clearly shown to be a significant risk factor for SARS-CoV-2 infection or poor outcomes in COVID-19, major modification to established guideline-recommended standard asthma treatment during the COVID-19 pandemic has not been deemed necessary. According to the Global Initiative for Asthma (GINA) guidance about COVID-19 and asthma updated in March 2021, it is important to continue good asthma management in order to maintain optimal symptom control, to reduce the risk of severe exacerbations, and to minimize the need for oral corticosteroids, thus reducing the need to seek urgent medical care and consequential potential exposure to SARS-CoV-2 [47]. In a nationwide health insurance claims data-based study conducted in Korea, which included 218 patients with confirmed COVID-19 and underlying asthma, in univariate analyses, use of short-acting beta agonists was a significant risk factor for intensive care unit admission and use of long-acting beta agonists was a significant protective factor for hospital admission duration, though these factors were no longer significant in multivariate analyses [48]. In terms of the total medical cost burden associated with COVID-19, use of oral short-acting beta agonists in the past year was an independent risk factor for increased cost burden. Furthermore, compared to patients with GINA step 1 asthma (mild asthma not requiring maintenance treatment), patients with GINA step 5 asthma (moderate-to-severe asthma that is difficult to control despite a medium/high dose inhaled corticosteroid plus long-acting beta agonist) required a longer duration of hospitalization for COVID-19 in both univariate and multivariate analyses. These findings support that optimal asthma control with an adequate use of maintenance therapies may improve prognosis in patients with asthma who contract COVID-19; therefore, patients should be advised to continue taking their prescribed asthma medications, including biologic therapy and inhaled or oral corticosteroids, as is recommended by GINA. In addition, all patients should have a written asthma action plan that advises on controller and reliever medication use in case of worsening asthma symptoms. In addition, GINA guidance recommends the avoidance of nebulizer use as a precaution against virus transmission via airborne particles, and that switching to pressurized metered dose inhalers (with a spacer if needed) is preferable. Similarly, avoidance of spirometry during healthcare visits is also recommended as a transmission-based precaution.

## 6. COVID-19 Vaccination in Patients with Asthma

In addition to maintaining optimal symptom control by continuing all prescribed asthma medications during the pandemic, patients with asthma should be further protected with COVID-19 vaccination. Patients with chronic allergic and atopic diseases, including those treated with biologic agents, have not exhibited increased risk for hypersensitivity reactions after a COVID-19 vaccination [49]. Based on presently understood risks and benefits, GINA guidance recommends that people with asthma undergo COVID-19 vaccination, and the Pfizer/BioNTech and Moderna COVID-19 vaccines were specifically mentioned in the GINA guidance document released in October 2021 [47]. The safety and tolerability of the mRNA COVID-19 Pfizer/BioNTech vaccine in 253 patients with severe asthma were evaluated in a survey study conducted in Italy [50]. According to patient-reported results collected via a vaccination-related adverse events questionnaire, over 80% of patients did not report adverse events after receiving the first and second doses of the vaccine. Among patients who did report experiencing adverse events following vaccination, the reported effects were mostly very common effects such as injection site pain and swelling, weakness, fever, myalgia, arthralgia, and headache, reported by 80% of patients after the first dose and 95% after the second dose, and no patient reported experiencing a rare post-vaccination side effect, including facial asymmetry or severe allergic reaction. In this population of severe asthma patients, 220 (87%) patients were receiving ongoing biologic treatment, and proportions of patients experiencing adverse events following the first and second vaccine doses were comparable across biologic agents (benralizumab 16.9–23.1%, mepolizumab 19.3–20.7%, omalizumab 21.8–22.8%, dupilumab 11.1%). According to GINA guidance, patients with asthma should continue to receive the annual influenza vaccine, with a separation of at least 14 days between COVID-19 and influenza vaccinations. Finally, guidance suggests that biologic therapy and a COVID-19 vaccine should not be given on the same day to allow adverse effects of either to be more easily distinguishable.

## 7. Impact of Oral and Inhaled Corticosteroids on COVID-19

Compared to patients with asthma of Th2-low endotype, those with Th2-high endotype show higher responsiveness to corticosteroid treatment [51]. As corticosteroids have immunosuppressive effects, the impact of corticosteroids on COVID-19 outcomes may be concerning for many clinicians [52]. Previous studies of corticosteroid treatment during respiratory viral illness, including SARS, have generally shown a lack of effectiveness in reducing morbidity and instead, possible harm [53,54]. Earlier in the pandemic, when COVID-19-specific evidence relating to corticosteroid use was still largely lacking, the World Health Organization (WHO) initially recommended against the use of systemic corticosteroids to treat COVID-19 in its clinical management guidance document released in March 2020 [55]. However, as studies evaluating various treatments, including corticosteroids, in patients with COVID-19 have rapidly accumulated, the WHO changed its stance in a guidance document released in September 2020, which stated a strong recommendation for the use of systemic corticosteroids in the treatment of patients with severe and critical COVID-19 based on the most current evidence [56]. The updated WHO guidance specifically referenced the preliminary report of the now published RECOVERY multicenter, randomized controlled trial, which included 6425 hospitalized patients with COVID-19 who were randomized to receive oral or intravenous dexamethasone 6 mg once daily in addition to usual care or usual care alone [57]. Final results of the RECOVERY study showed that dexamethasone-treated COVID-19 patients had a significantly lower risk of the primary outcome of 28-day mortality compared to those who received usual care alone (age-adjusted rate ratio, 0.83; 95% CI, 0.75–0.93; *p* < 0.001) [57]. However, in the subgroup analysis according to respiratory support at randomization, mortality risk was significantly reduced in patients requiring invasive mechanical ventilation or oxygen only, but was not significantly reduced in patients who did not require respiratory support. In the PRINCIPLE study [58], which is a randomized controlled trial, older non-hospitalized patients with COVID-19 (65 years of older or 50 years or older with comorbidities) who received inhaled budesonide 800 µg twice daily for 14 days in addition to usual care had a significantly shorter time to recovery by approximately 3 days compared to those who received usual care alone.

The burden of COVID-19 among patients with chronic respiratory diseases has not been shown to be increased, and indeed was shown to be decreased in some studies, compared to the general population [59]. This rather unexpected finding has been hypothesized to be attributed to potential protective effects of respiratory disease treatments against SARS-CoV-2 infection and severe disease. Observational studies have shown that inhaled corticosteroid use in patients with asthma was associated with the increased prevalence of non-COVID-19 upper and lower respiratory tract infections [60,61]. In contrast, inhaled corticosteroid treatment has been associated with a reduced expression of ACE2, the entryway of SARS-CoV-2 into host cells, thereby potentially reducing SARS-CoV-2 susceptibility and morbidity [62,63]. Results of an in vitro study have shown that pre-treatment of human nasal and tracheal epithelial cells with budesonide, glycopyrronium, and formoterol inhibited coronavirus HCoV-229E replication and cytokine production [64]. An in vitro study has revealed that the corticosteroids mometasone and ciclesonide suppressed replication of SARS-CoV-2 in a culture medium of infected cells, and that ciclesonide was particularly effective in a concentration-dependent manner [65]. Furthermore, a study that screened a panel of 48 United States Food and Drug Administration-approved drugs identified ciclesonide as an inhibitor of SARS-CoV-2 cytopathic viral activity [66]. In addition to the inhibitory effects on viral replication, treatment with corticosteroids might also reduce the severity of COVID-19 by inhibiting virus-induced cytokine release and dampening the exaggerated inflammatory response responsible for severe symptoms [67]. Overall, the effects of the appropriate use of oral and inhaled corticosteroids is expected to lean toward being beneficial rather than harmful for patients with asthma during the pandemic, and adherence to all maintenance asthma medications to ensure good symptom control and to prevent exacerbation should be emphasized, as is consistently recommended by professional organizations worldwide [68].

## 8. Role of IgE in the Response to Respiratory Viral Infection

Robust evidence supports the impairment of antiviral response and occurrence of the exacerbation-inducing effect of viral infection in patients with atopic diseases such as asthma [69,70,71,72,73]. Inflammatory mediators released in allergic inflammation result in epithelial barrier disruption and airway remodeling, which may increase susceptibility to respiratory viral infection, and viral infection further induces pro-inflammatory cytokine activity [74,75]. Moreover, atopic asthma with allergic sensitization has been associated with reduced viral clearance, reduced virus-induced IFN responses, and increased viral shedding [18]. Early after respiratory virus infection, myriad cytokines and chemokines activate and attract mast cells, dendritic cells, granulocytes, and monocytes at the infection site [76,77]. Although mast cells are important players in the front-line defense against antigens, the acute hypersensitivity reaction caused by the overactivation of mast cells in highly pathogenic viral infections can be detrimental [77]. In the respiratory tract, mast cell degranulation increases vascular permeability, edema, and mucus production, which lead to airway constriction, congestion, and cough [78,79,80]. Binding of antigen-specific IgE to the high-affinity IgE receptor (FcɛRI) activates mast cells, enhances mast cell survival, and sensitizes mast cells to subsequent encounters with the antigen [81]. Notably, high concentrations of free IgE has been shown to activate mast cells similarly to antigen-bound IgE, thus propagating the immune response even in the absence of antigen stimulation [82]. The anti-IgE therapy omalizumab, which impedes IgE-dependent cellular events by binding to free IgE, prevents binding of IgE to FcɛRI, and reduces the expression of FcɛRI, was the first biologic therapy to be licensed as an add-on treatment for patients with moderate-to-severe asthma [14].

**Figure 2:**
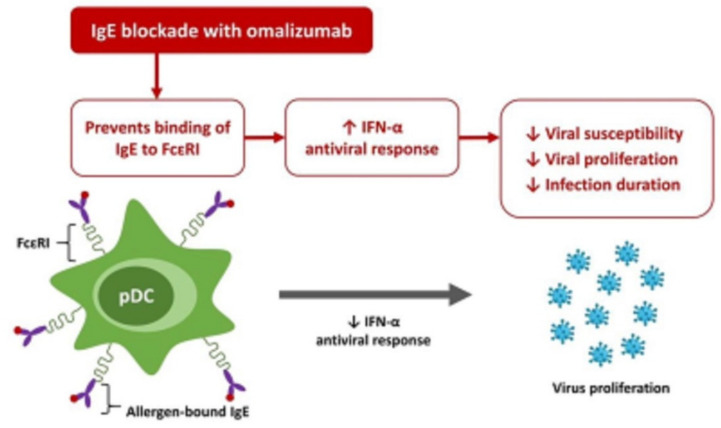
Role of IgE and effects of IgE blockade with omalizumab in antiviral response. Cross-linking of IgE bound to FcɛRI on pDCs, which are mainly located in lung interstitium, diminishes IFN-α antiviral response. Anti-IgE therapy omalizumab binds to free IgE, prevents binding of IgE to FcɛRI, and reduces the expression of FcɛRI, thereby increases IFN-α antiviral response. FcɛRI, high-affinity IgE receptor; IFN-α, interferon-α; IgE, immunoglobulin E; and pDC, plasmacytoid dendritic cell.

In the immune response to acute viral infections, plasmacytoid dendritic cells recruited to the respiratory tract produce large quantities of type I IFN, mediated by toll-like receptors, which stimulates T cells and drives antiviral responses (Figure 2) [83]. In a case-control study of patients with allergic asthma, when exposed to the virus, plasmacytoid dendritic cells purified from the whole blood of patients with allergic asthma exhibited significantly reduced expression of IFN-α compared to the plasmacytoid dendritic cells of healthy controls, suggesting a delayed and inefficient antiviral immune response [71]. Expression of FcɛRI on plasmacytoid dendritic cells was significantly increased in patients with allergic asthma compared to controls, and both FcɛRI expression and serum IgE concentration were significantly inversely correlated with IFN-α secretion upon viral exposure. Importantly, the cross-linking of IgE bound to FcɛRI was found to diminish the IFN-α antiviral response of plasmacytoid dendritic cells in a dose-dependent manner. Therefore, findings suggest that elevated serum IgE level and FcɛRI expression are associated with an excessive inflammatory response to viral infection as well as a weakened antiviral response. Furthermore, respiratory viruses are known to evade innate type 1 immunity to increase the opportunity for viral replication, which in turn promotes a stronger and more sustained subsequent type 2 inflammatory response [84,85,86].

The critical role of IgE in instigating the inflammatory response to respiratory viral infection has been further supported by the results of multiple randomized trials [87,88,89,90,91]. Notably, studies have demonstrated a reduction in viral infection-induced exacerbations in pediatric asthma patients treated with omalizumab. In the landmark PROSE (Preventative Omalizumab or Step-up Therapy for Severe Fall Exacerbations) study, 478 urban school-aged children with allergic asthma were randomized to receive placebo or omalizumab as an add-on treatment to guideline-based standard asthma care [89,90]. Compared with placebo, treatment with omalizumab significantly decreased the risk of respiratory virus-associated exacerbations in children with severe persistent asthma (OR, 0.35; 95% CI, 0.15–0.85). Ex vivo experiments using nasal mucus samples obtained from participants in this study showed that in the presence of IgE cross-linking, omalizumab significantly increased the IFN-α response to rhinovirus by over three-fold compared to placebo (*p* = 0.03) [90]. Further experiments carried out on the nasal mucus samples showed that omalizumab significantly decreased weekly rhinovirus detection rates (OR, 0.74; 95% CI, 0.60–0.92), indicating a reduced duration of viral infection, and significantly reduced peak viral shedding (*p* < 0.04), suggesting decreased severity of viral illness [89]. These ex vivo results were echoed in the significant reduction in the frequency of rhinovirus illness observed with omalizumab compared with placebo (risk ratio, 0.64; 95% CI, 0.49–0.84). These findings provide strong support that blocking IgE reduces the frequency, duration, and severity of respiratory viral illness in patients with asthma.

## 9. Anti-IgE Biologic Agent as a Potential Treatment for COVID-19

In the majority of people infected with SARS-CoV-2, the infection follows a mild to moderate self-limiting course that is not much different from the typical course of common respiratory virus infections [92,93,94]. In a subset of patients, however, SARS-CoV-2 infection evokes a hyperinflammatory immune response characterized by an exaggerated increase in the release of cytokines (IL-2, IL-6, IL-7, IL-10, IL-12, TNF-α, CXCL10, CCL2, CCL3), aptly known as “cytokine storm”, and the disproportionate inflammatory activation may lead to acute respiratory distress, multi-organ failure, and possible death [92,95]. Considering the well-established role of IgE in atopic diseases, airway hyperreactivity, and antiviral response, an investigation of anti-IgE as a potential treatment to COVID-19 is warranted. Among the currently available biologic treatments for severe asthma, omalizumab is the only agent with a documented effect in viral respiratory infections [95]. The protective action of omalizumab against viral infections is multifaceted. Omalizumab directly reduces levels of free IgE, inhibits cross-linking of IgE-bound FcɛRI, and inhibits mast cell activation that can trigger and propagate the hyperinflammatory cascade (Figure 2) [89]. In addition, omalizumab indirectly reduces the number of FcɛRI on basophils, mast cells, and dendritic cells, which helps to prevent the hindrance of an IFN-α-mediated antiviral response [95]. Conversely, as eosinophils have potential antiviral activity, anti-eosinophilic biologic agents might theoretically be less beneficial in COVID-19, though supporting clinical evidence is currently sparse [96]. In a case report of a 41-year-old male patient with severe eosinophilic asthma and a 2-year history of treatment with benralizumab, an anti-IL-5 receptor agent with eosinophil-depleting activity, the patient experienced a mild course of COVID-19 without a marked loss of asthma control [97].

Although still limited in number, reports describing the use of omalizumab in asthma patients during the COVID-19 pandemic are accumulating [98,99,100]. Lommatzsch et al. reported the results of a 52-year-old German male patient with severe early-onset allergic asthma treated with omalizumab for 6 months who was infected with SARS-CoV-2 [98]. The patient did not experience dyspnea, worsening of asthma, or need for short-acting bronchodilator therapy during the course of infection, and omalizumab treatment was not interrupted. A study conducted in Turkey that surveyed 75 patients with severe asthma treated with omalizumab or mepolizumab revealed that compared to patients who continued biologic treatment, risk of COVID-19 was significantly higher in the 12 patients who interrupted biologic treatment due to a refusal to return to the hospital during the pandemic (relative risk, 2.71; 95% CI, 1.21–6.06) [100]. Another Turkish study showed that among 13 patients with severe asthma treated with omalizumab or mepolizumab who had contracted SARS-CoV-2, five (38.5%) had mild COVID-19, eight (61.5%) had moderate COVID-19, none suffered severe COVID-19 requiring mechanical ventilation or intensive care, and all patients fully recovered [99]. These findings provide reassurance that omalizumab may be safely continued during active COVID-19 infection and may potentially reduce the risk of severe COVID-19 in patients with asthma. Furthermore, maintaining good control of asthma with continued biologic treatment during the pandemic at least reduces the risk of exacerbations requiring medical care and thus reduces the risk of exposure to SARS-CoV-2.

## 10. Conclusions

Growing evidence reassuringly supports that asthma and asthma treatments do not seem to increase the risk of SARS-CoV-2 infection or severe COVID-19 illness. Indeed, patients with the Th2-high endotype of asthma may be at lower risk for COVID-19 due to increased eosinophil levels and reduced ACE2 expression. Patients should be urged to continue all prescribed asthma medications, including inhaled corticosteroids and biologic treatment, and to receive full COVID-19 vaccination. Th2-high endotype patients treated with omalizumab might be further protected from COVID-19, as the role of IgE in reducing an antiviral response and the beneficial effects of an anti-IgE treatment on immune responses against viral infection are well established. The impact of the different biologic treatments on COVID-19 will be better understood as more evidence emerges. Finally, omalizumab as a potential treatment for COVID-19 warrants investigation. 

## Data Availability

Not applicable.

## References

[1] Cao Y., Liu X., Xiong L., Cai K. (2020). Imaging and clinical features of patients with 2019 novel coronavirus SARS-CoV-2: A systematic review and meta-analysis. J. Med. Virol..

[2] Liu S., Zhi Y., Ying S. (2020). COVID-19 and Asthma: Reflection During the Pandemic. Clin. Rev. Allergy Immunol..

[3] Broadhurst R., Peterson R., Wisnivesky J.P., Federman A., Zimmer S.M., Sharma S., Wechsler M., Holguin F. (2020). Asthma in COVID-19 Hospitalizations: An Overestimated Risk Factor?. Ann. Am. Thorac. Soc..

[4] Grandbastien M., Piotin A., Godet J., Abessolo-Amougou I., Ederle C., Enache I., Fraisse P., Tu Hoang T.C., Kassegne L., Labani A. (2020). SARS-CoV-2 Pneumonia in Hospitalized Asthmatic Patients Did Not Induce Severe Exacerbation. J. Allergy Clin. Immunol. Pract..

[5] Wang Y., Chen J., Chen W., Liu L., Dong M., Ji J., Hu D., Zhang N. (2021). Does Asthma Increase the Mortality of Patients with COVID-19?: A Systematic Review and Meta-Analysis. Int. Arch. Allergy Immunol..

[6] Ferastraoaru D., Hudes G., Jerschow E., Jariwala S., Karagic M., de Vos G., Rosenstreich D., Ramesh M. (2021). Eosinophilia in Asthma Patients Is Protective Against Severe COVID-19 Illness. J. Allergy Clin. Immunol. Pract..

[7] Jackson D.J., Busse W.W., Bacharier L.B., Kattan M., O’Connor G.T., Wood R.A., Visness C.M., Durham S.R., Larson D., Esnault S. (2020). Association of respiratory allergy, asthma, and expression of the SARS-CoV-2 receptor ACE2. J. Allergy Clin. Immunol..

[8] Busse W.W. (2019). Biological treatments for severe asthma: A major advance in asthma care. Allergol. Int..

[9] Kuruvilla M.E., Lee F.E., Lee G.B. (2019). Understanding Asthma Phenotypes, Endotypes, and Mechanisms of Disease. Clin. Rev. Allergy Immunol..

[10] Busse W.W., Kraft M., Rabe K.F., Deniz Y., Rowe P.J., Ruddy M., Castro M. (2021). Understanding the key issues in the treatment of uncontrolled persistent asthma with type 2 inflammation. Eur. Respir. J..

[11] Miranda C., Busacker A., Balzar S., Trudeau J., Wenzel S.E. (2004). Distinguishing severe asthma phenotypes: Role of age at onset and eosinophilic inflammation. J. Allergy Clin. Immunol..

[12] Wenzel S.E., Schwartz L.B., Langmack E.L., Halliday J.L., Trudeau J.B., Gibbs R.L., Chu H.W. (1999). Evidence that severe asthma can be divided pathologically into two inflammatory subtypes with distinct physiologic and clinical characteristics. Am. J. Respir. Crit. Care Med..

[13] Svenningsen S., Nair P. (2017). Asthma Endotypes and an Overview of Targeted Therapy for Asthma. Front. Med..

[14] Chiu C.J., Huang M.T. (2021). Asthma in the Precision Medicine Era: Biologics and Probiotics. Int. J. Mol. Sci..

[15] Halim T.Y.F., McKenzie A.N.J. (2013). New kids on the block: Group 2 innate lymphoid cells and type 2 inflammation in the lung. Chest.

[16] Licona-Limon P., Kim L.K., Palm N.W., Flavell R.A. (2013). TH2, allergy and group 2 innate lymphoid cells. Nat. Immunol..

[17] James K.M., Peebles R.S., Hartert T.V. (2012). Response to infections in patients with asthma and atopic disease: An epiphenomenon or reflection of host susceptibility?. J. Allergy Clin. Immunol..

[18] Jartti T., Gern J.E. (2017). Role of viral infections in the development and exacerbation of asthma in children. J. Allergy Clin. Immunol..

[19] O’Riordan S., Barton M., Yau Y., Read S.E., Allen U., Tran D. (2010). Risk factors and outcomes among children admitted to hospital with pandemic H1N1 influenza. CMAJ.

[20] Center for Disease Control and Preventions Coronavirus 19. https://www.cdc.gov/coronavirus/2019-ncov/need-extra-precautions/asthma.html.

[21] Lombardi C., Gani F., Berti A., Comberiati P., Peroni D., Cottini M. (2021). Asthma and COVID-19: A dangerous liaison?. Asthma Res. Pract..

[22] Liu S., Cao Y., Du T., Zhi Y. (2021). Prevalence of Comorbid Asthma and Related Outcomes in COVID-19: A Systematic Review and Meta-Analysis. J. Allergy Clin. Immunol. Pract..

[23] Terry P.D., Heidel R.E., Dhand R. (2021). Asthma in Adult Patients with COVID-19. Prevalence and Risk of Severe Disease. Am. J. Respir. Crit. Care Med..

[24] Yang J.M., Koh H.Y., Moon S.Y., Yoo I.K., Ha E.K., You S., Kim S.Y., Yon D.K., Lee S.W. (2020). Allergic disorders and susceptibility to and severity of COVID-19: A nationwide cohort study. J. Allergy Clin. Immunol..

[25] Calmes D., Graff S., Maes N., Frix A.N., Thys M., Bonhomme O., Berg J., Debruche M., Gester F., Henket M. (2021). Asthma and COPD Are Not Risk Factors for ICU Stay and Death in Case of SARS-CoV2 Infection. J. Allergy Clin. Immunol. Pract..

[26] Zhu Z., Hasegawa K., Ma B., Fujiogi M., Camargo C.A., Liang L. (2020). Association of asthma and its genetic predisposition with the risk of severe COVID-19. J. Allergy Clin. Immunol..

[27] Beurnier A., Jutant E.M., Jevnikar M., Boucly A., Pichon J., Preda M., Frank M., Laurent J., Richard C., Monnet X. (2020). Characteristics and outcomes of asthmatic patients with COVID-19 pneumonia who require hospitalisation. Eur. Respir. J..

[28] Carli G., Cecchi L., Stebbing J., Parronchi P., Farsi A. (2021). Is asthma protective against COVID-19?. Allergy.

[29] Rosenberg H.F., Dyer K.D., Domachowske J.B. (2009). Respiratory viruses and eosinophils: Exploring the connections. Antiviral Res..

[30] Chen D., Zhang S., Feng Y., Wu W., Chang C., Chen S., Zhen G., Yi L. (2021). Decreased eosinophil counts and elevated lactate dehydrogenase predict severe COVID-19 in patients with underlying chronic airway diseases. Postgrad. Med. J..

[31] Ackermann M., Anders H.J., Bilyy R., Bowlin G.L., Daniel C., De Lorenzo R., Egeblad M., Henneck T., Hidalgo A., Hoffmann M. (2021). Patients with COVID-19: In the dark-NETs of neutrophils. Cell Death Differ..

[32] Basnet S., Palmenberg A.C., Gern J.E. (2019). Rhinoviruses and Their Receptors. Chest.

[33] Bochkov Y.A., Gern J.E. (2016). Rhinoviruses and Their Receptors: Implications for Allergic Disease. Curr. Allergy Asthma Rep..

[34] Hoffmann M., Kleine-Weber H., Schroeder S., Kruger N., Herrler T., Erichsen S., Schiergens T.S., Herrler G., Wu N.H., Nitsche A. (2020). SARS-CoV-2 Cell Entry Depends on ACE2 and TMPRSS2 and Is Blocked by a Clinically Proven Protease Inhibitor. Cell.

[35] Ni W., Yang X., Yang D., Bao J., Li R., Xiao Y., Hou C., Wang H., Liu J., Yang D. (2020). Role of angiotensin-converting enzyme 2 (ACE2) in COVID-19. Crit. Care.

[36] Rath S., Perikala V., Jena A.B., Dandapat J. (2021). Factors regulating dynamics of angiotensin-converting enzyme-2 (ACE2), the gateway of SARS-CoV-2: Epigenetic modifications and therapeutic interventions by epidrugs. Biomed. Pharmacother..

[37] Kimura H., Francisco D., Conway M., Martinez F.D., Vercelli D., Polverino F., Billheimer D., Kraft M. (2020). Type 2 inflammation modulates ACE2 and TMPRSS2 in airway epithelial cells. J. Allergy Clin. Immunol..

[38] Camiolo M., Gauthier M., Kaminski N., Ray A., Wenzel S.E. (2020). Expression of SARS-CoV-2 receptor ACE2 and coincident host response signature varies by asthma inflammatory phenotype. J. Allergy Clin. Immunol..

[39] Song J., Zeng M., Wang H., Qin C., Hou H.Y., Sun Z.Y., Xu S.P., Wang G.P., Guo C.L., Deng Y.K. (2021). Distinct effects of asthma and COPD comorbidity on disease expression and outcome in patients with COVID-19. Allergy.

[40] Alashkar Alhamwe B., Miethe S., Pogge von Strandmann E., Potaczek D.P., Garn H. (2020). Epigenetic Regulation of Airway Epithelium Immune Functions in Asthma. Front. Immunol..

[41] Bridges J.P., Vladar E.K., Huang H., Mason R.J. (2022). Respiratory epithelial cell responses to SARS-CoV-2 in COVID-19. Thorax.

[42] Lopez J., Mommert M., Mouton W., Pizzorno A., Brengel-Pesce K., Mezidi M., Villard M., Lina B., Richard J.C., Fassier J.B. (2021). Early nasal type I IFN immunity against SARS-CoV-2 is compromised in patients with autoantibodies against type I IFNs. J. Exp. Med..

[43] Pizzorno A., Padey B., Julien T., Trouillet-Assant S., Traversier A., Errazuriz-Cerda E., Fouret J., Dubois J., Gaymard A., Lescure F.X. (2020). Characterization and Treatment of SARS-CoV-2 in Nasal and Bronchial Human Airway Epithelia. Cell Rep. Med..

[44] Zhu N., Wang W., Liu Z., Liang C., Wang W., Ye F., Huang B., Zhao L., Wang H., Zhou W. (2020). Morphogenesis and cytopathic effect of SARS-CoV-2 infection in human airway epithelial cells. Nat. Commun..

[45] Potaczek D.P., Miethe S., Schindler V., Alhamdan F., Garn H. (2020). Role of airway epithelial cells in the development of different asthma phenotypes. Cell. Signal..

[46] Alashkar Alhamwe B., Potaczek D.P., Miethe S., Alhamdan F., Hintz L., Magomedov A., Garn H. (2021). Extracellular Vesicles and Asthma-More Than Just a Co-Existence. Int. J. Mol. Sci..

[47] Global Initiative for Asthma GINA Guidance about COVID-19 and Asthma. Updated 30 March 2021. https://ginasthma.org/wp-content/uploads/2021/03/21_03_30-GINA-COVID-19-and-asthma.pdf.

[48] Choi Y.J., Park J.Y., Lee H.S., Suh J., Song J.Y., Byun M.K., Cho J.H., Kim H.J., Lee J.H., Park J.W. (2021). Effect of asthma and asthma medication on the prognosis of patients with COVID-19. Eur. Respir. J..

[49] Pfaar O., Klimek L., Hamelmann E., Kleine-Tebbe J., Taube C., Wagenmann M., Werfel T., Brehler R., Novak N., Mulleneisen N. (2021). COVID-19 vaccination of patients with allergies and type-2 inflammation with concurrent antibody therapy (biologicals)—A Position Paper of the German Society of Allergology and Clinical Immunology (DGAKI) and the German Society for Applied Allergology (AeDA). Allergol. Select.

[50] Caminati M., Guarnieri G., Batani V., Scarpieri E., Finocchiaro A., Chieco-Bianchi F., Senna G., Vianello A. (2021). COVID-19 Vaccination in Patients with Severe Asthma on Biologic Treatment: Safety, Tolerability, and Impact on Disease Control. Vaccines.

[51] Ramadan A.A., Gaffin J.M., Israel E., Phipatanakul W. (2019). Asthma and Corticosteroid Responses in Childhood and Adult Asthma. Clin. Chest Med..

[52] FakhriRavari A., Jin S., Kachouei F.H., Le D., Lopez M. (2021). Systemic corticosteroids for management of COVID-19: Saving lives or causing harm?. Int. J. Immunopathol. Pharmacol..

[53] Russell C.D., Millar J.E., Baillie J.K. (2020). Clinical evidence does not support corticosteroid treatment for 2019-nCoV lung injury. Lancet.

[54] Stockman L.J., Bellamy R., Garner P. (2006). SARS: Systematic review of treatment effects. PLoS Med..

[55] World Health Organization (2020). Clinical Management of Severe Acute Respiratory Infection (SARI) When COVID-19 Disease is Suspected. Interim Guidance. https://www.who.int/docs/default-source/coronaviruse/clinical-managementof-novel-cov.pdf.

[56] World Health Organization Corticosteroids for COVID-19. Living Guidance, 2 September 2020. https://www.who.int/publications/i/item/WHO-2019-nCoV-Corticosteroids-2020.1.

[57] Horby P., Lim W.S., Emberson J.R., Mafham M., Bell J.L., Linsell L., Staplin N., Brightling C., Ustianowski A., Elmahi E. (2021). Dexamethasone in Hospitalized Patients with COVID-19. N. Engl. J. Med..

[58] Yu L.M., Bafadhel M., Dorward J., Hayward G., Saville B.R., Gbinigie O., Van Hecke O., Ogburn E., Evans P.H., Thomas N.P.B. (2021). Inhaled budesonide for COVID-19 in people at high risk of complications in the community in the UK (PRINCIPLE): A randomised, controlled, open-label, adaptive platform trial. Lancet.

[59] Halpin D.M.G., Faner R., Sibila O., Badia J.R., Agusti A. (2020). Do chronic respiratory diseases or their treatment affect the risk of SARS-CoV-2 infection?. Lancet Respir. Med..

[60] McKeever T., Harrison T.W., Hubbard R., Shaw D. (2013). Inhaled corticosteroids and the risk of pneumonia in people with asthma: A case-control study. Chest.

[61] Yang M., Chen H., Zhang Y., Du Y., Xu Y., Jiang P., Xu Z. (2017). Long-term use of inhaled corticosteroids and risk of upper respiratory tract infection in chronic obstructive pulmonary disease: A meta-analysis. Inhal. Toxicol..

[62] Beyerstedt S., Casaro E.B., Rangel E.B. (2021). COVID-19: Angiotensin-converting enzyme 2 (ACE2) expression and tissue susceptibility to SARS-CoV-2 infection. Eur. J. Clin. Microbiol. Infect. Dis..

[63] Peters M.C., Sajuthi S., Deford P., Christenson S., Rios C.L., Montgomery M.T., Woodruff P.G., Mauger D.T., Erzurum S.C., Johansson M.W. (2020). COVID-19-related Genes in Sputum Cells in Asthma. Relationship to Demographic Features and Corticosteroids. Am. J. Respir. Crit. Care Med..

[64] Yamaya M., Nishimura H., Deng X., Sugawara M., Watanabe O., Nomura K., Shimotai Y., Momma H., Ichinose M., Kawase T. (2020). Inhibitory effects of glycopyrronium, formoterol, and budesonide on coronavirus HCoV-229E replication and cytokine production by primary cultures of human nasal and tracheal epithelial cells. Respir. Investig..

[65] Matsuyama S., Kawase M., Nao N., Shirato K., Ujike M., Kamitani W., Shimojima M., Fukushi S. (2020). The Inhaled Steroid Ciclesonide Blocks SARS-CoV-2 RNA Replication by Targeting the Viral Replication-Transcription Complex in Cultured Cells. J. Virol..

[66] Jeon S., Ko M., Lee J., Choi I., Byun S.Y., Park S., Shum D., Kim S. (2020). Identification of Antiviral Drug Candidates against SARS-CoV-2 from FDA-Approved Drugs. Antimicrob. Agents Chemother..

[67] Oliver B.G., Robinson P., Peters M., Black J. (2014). Viral infections and asthma: An inflammatory interface?. Eur. Respir. J..

[68] Ong K.Y., Tan T.L., Chan A.K.W., Tan K.L.L., Koh M.S. (2021). Managing asthma in the COVID-19 pandemic and current recommendations from professional bodies: A review. J. Asthma.

[69] Duff A.L., Pomeranz E.S., Gelber L.E., Price G.W., Farris H., Hayden F.G., Platts-Mills T.A., Heymann P.W. (1993). Risk factors for acute wheezing in infants and children: Viruses, passive smoke, and IgE antibodies to inhalant allergens. Pediatrics.

[70] Gern J.E. (2015). Virus/Allergen Interaction in Asthma Exacerbation. Ann. Am. Thorac. Soc..

[71] Gill M.A., Bajwa G., George T.A., Dong C.C., Dougherty I.I., Jiang N., Gan V.N., Gruchalla R.S. (2010). Counterregulation between the FcepsilonRI pathway and antiviral responses in human plasmacytoid dendritic cells. J. Immunol..

[72] Lynch J.P., Werder R.B., Simpson J., Loh Z., Zhang V., Haque A., Spann K., Sly P.D., Mazzone S.B., Upham J.W. (2016). Aeroallergen-induced IL-33 predisposes to respiratory virus-induced asthma by dampening antiviral immunity. J. Allergy Clin. Immunol..

[73] Soto-Quiros M., Avila L., Platts-Mills T.A., Hunt J.F., Erdman D.D., Carper H., Murphy D.D., Odio S., James H.R., Patrie J.T. (2012). High titers of IgE antibody to dust mite allergen and risk for wheezing among asthmatic children infected with rhinovirus. J. Allergy Clin. Immunol..

[74] Georas S.N., Rezaee F. (2014). Epithelial barrier function: At the front line of asthma immunology and allergic airway inflammation. J. Allergy Clin. Immunol..

[75] Jackson D.J., Johnston S.L. (2010). The role of viruses in acute exacerbations of asthma. J. Allergy Clin. Immunol..

[76] Rossi G.A., Colin A.A. (2015). Infantile respiratory syncytial virus and human rhinovirus infections: Respective role in inception and persistence of wheezing. Eur. Respir. J..

[77] Graham A.C., Temple R.M., Obar J.J. (2015). Mast cells and influenza a virus: Association with allergic responses and beyond. Front. Immunol..

[78] Bradding P. (1999). Allergen immunotherapy and mast cells. Clin. Exp. Allergy.

[79] Da Silva E.Z., Jamur M.C., Oliver C. (2014). Mast cell function: A new vision of an old cell. J. Histochem. Cytochem..

[80] Hofmann A.M., Abraham S.N. (2009). New roles for mast cells in modulating allergic reactions and immunity against pathogens. Curr. Opin. Immunol..

[81] Galli S.J., Tsai M. (2012). IgE and mast cells in allergic disease. Nat. Med..

[82] Kawakami T., Kitaura J. (2005). Mast cell survival and activation by IgE in the absence of antigen: A consideration of the biologic mechanisms and relevance. J. Immunol..

[83] Cella M., Facchetti F., Lanzavecchia A., Colonna M. (2000). Plasmacytoid dendritic cells activated by influenza virus and CD40L drive a potent TH1 polarization. Nat. Immunol..

[84] Holt P.G., Sly P.D. (2012). Viral infections and atopy in asthma pathogenesis: New rationales for asthma prevention and treatment. Nat. Med..

[85] Subrata L.S., Bizzintino J., Mamessier E., Bosco A., McKenna K.L., Wikstrom M.E., Goldblatt J., Sly P.D., Hales B.J., Thomas W.R. (2009). Interactions between innate antiviral and atopic immunoinflammatory pathways precipitate and sustain asthma exacerbations in children. J. Immunol..

[86] Kikkert M. (2020). Innate Immune Evasion by Human Respiratory RNA Viruses. J. Innate Immun..

[87] Busse W.W., Morgan W.J., Gergen P.J., Mitchell H.E., Gern J.E., Liu A.H., Gruchalla R.S., Kattan M., Teach S.J., Pongracic J.A. (2011). Randomized trial of omalizumab (anti-IgE) for asthma in inner-city children. N. Engl. J. Med..

[88] Heymann P.W., Platts-Mills T.A.E., Woodfolk J.A., Borish L., Murphy D.D., Carper H.T., Conaway M.R., Steinke J.W., Muehling L., Gerald Teague W. (2020). Understanding the asthmatic response to an experimental rhinovirus infection: Exploring the effects of blocking IgE. J. Allergy Clin. Immunol..

[89] Esquivel A., Busse W.W., Calatroni A., Togias A.G., Grindle K.G., Bochkov Y.A., Gruchalla R.S., Kattan M., Kercsmar C.M., Khurana Hershey G. (2017). Effects of Omalizumab on Rhinovirus Infections, Illnesses, and Exacerbations of Asthma. Am. J. Respir. Crit. Care Med..

[90] Teach S.J., Gill M.A., Togias A., Sorkness C.A., Arbes S.J., Calatroni A., Wildfire J.J., Gergen P.J., Cohen R.T., Pongracic J.A. (2015). Preseasonal treatment with either omalizumab or an inhaled corticosteroid boost to prevent fall asthma exacerbations. J. Allergy Clin. Immunol..

[91] Pelaia C., Calabrese C., Terracciano R., de Blasio F., Vatrella A., Pelaia G. (2018). Omalizumab, the first available antibody for biological treatment of severe asthma: More than a decade of real-life effectiveness. Ther. Adv. Respir. Dis..

[92] Azkur A.K., Akdis M., Azkur D., Sokolowska M., van de Veen W., Bruggen M.C., O’Mahony L., Gao Y., Nadeau K., Akdis C.A. (2020). Immune response to SARS-CoV-2 and mechanisms of immunopathological changes in COVID-19. Allergy.

[93] Huang C., Wang Y., Li X., Ren L., Zhao J., Hu Y., Zhang L., Fan G., Xu J., Gu X. (2020). Clinical features of patients infected with 2019 novel coronavirus in Wuhan, China. Lancet.

[94] Wang D., Hu B., Hu C., Zhu F., Liu X., Zhang J., Wang B., Xiang H., Cheng Z., Xiong Y. (2020). Clinical Characteristics of 138 Hospitalized Patients With 2019 Novel Coronavirus-Infected Pneumonia in Wuhan, China. JAMA.

[95] Menzella F., Ghidoni G., Galeone C., Capobelli S., Scelfo C., Facciolongo N.C. (2021). Immunological Aspects Related to Viral Infections in Severe Asthma and the Role of Omalizumab. Biomedicines.

[96] Renner A., Marth K., Patocka K., Idzko M., Pohl W. (2020). COVID-19 in two severe asthmatics receiving benralizumab: Busting the eosinophilia myth. ERJ Open Res..

[97] Renner A., Marth K., Patocka K., Pohl W. (2021). COVID-19 in a severe eosinophilic asthmatic receiving benralizumab—A case study. J. Asthma.

[98] Lommatzsch M., Stoll P., Virchow J.C. (2020). COVID-19 in a patient with severe asthma treated with Omalizumab. Allergy.

[99] Aksu K., Yesilkaya S., Topel M., Turkyilmaz S., Ercelebi D.C., Oncul A., Kalkan I.K., Ates H. (2021). COVID-19 in a patient with severe asthma using mepolizumab. Allergy Asthma Proc..

[100] Tuncay G., Cakmak M.E., Can Bostan O., Kaya S.B., Damadoglu E., Karakaya G., Kalyoncu A.F. (2021). The course of COVID-19 in patients with severe asthma receiving biological treatment. J. Asthma.

